# Smartphone-Based Electrochemical Biosensor for On-Site Nutritional Quality Assessment of Coffee Blends

**DOI:** 10.3390/molecules28145425

**Published:** 2023-07-15

**Authors:** Cristine D’Agostino, Claudia Chillocci, Francesca Polli, Luca Surace, Federica Simonetti, Marco Agostini, Sergio Brutti, Franco Mazzei, Gabriele Favero, Rosaceleste Zumpano

**Affiliations:** 1 Department of Chemistry and Drug Technologies, Sapienza University of Rome, Piazzale Aldo Moro 5, 00185 Rome, Italy; cristine.dagostino@uniroma1.it (C.D.); claudia.chillocci@uniroma1.it (C.C.); francesca.polli@uniroma1.it (F.P.); luca.surace@uniroma1.it (L.S.); federica.simonetti@uniroma1.it (F.S.); marco.agostini@uniroma1.it (M.A.); franco.mazzei@uniroma1.it (F.M.); 2 Department of Chemistry, Sapienza University of Rome, Piazzale Aldo Moro 5, 00185 Rome, Italy; sergio.brutti@uniroma1.it; 3 Department of Environmental Biology, Sapienza University of Rome, Piazzale Aldo Moro 5, 00185 Rome, Italy

**Keywords:** polyphenols, smartphone-based electrochemical biosensors, TPP, amperometric biosensors, free radicals, coffee

## Abstract

This work aimed to develop an easy-to-use smartphone-based electrochemical biosensor to quickly assess a coffee blend’s total polyphenols (Phs) content at the industrial and individual levels. The device is based on a commercial carbon-based screen-printed electrode (SPE) modified with multi-walled carbon nanotubes (CNTs) and gold nanoparticles (GNPs). At the same time, the biological recognition element, Laccase from *Trametes versicolor*, *Tv*Lac, was immobilized on the sensor surface by using glutaraldehyde (GA) as a cross-linking agent. The platform was electrochemically characterized to ascertain the influence of the SPE surface modification on its performance. The working electrode (WE) surface morphology characterization was obtained by scanning electron microscopy (SEM) and Fourier-transform infrared (FT-IR) imaging. All the measurements were carried out with a micro-potentiostat, the Sensit Smart by PalmSens, connected to a smartphone. The developed biosensor provided a sensitivity of 0.12 μA/μM, a linear response ranging from 5 to 70 μM, and a lower detection limit (LOD) of 2.99 μM. Afterward, the biosensor was tested for quantifying the total Phs content in coffee blends, evaluating the influence of both the variety and the roasting degree. The smartphone-based electrochemical biosensor’s performance was validated through the Folin–Ciocâlteu standard method.

## 1. Introduction

In recent years, the growing demand for devices detecting specific substances has led to looking for new technologies with quick response and detection characteristics. In this field, smartphone-based electrochemical biosensors are emerging as powerful tools for quickly detecting markers in healthcare, environmental monitoring, and food safety [1]. These biosensors can provide rapid, sensitive, and selective analysis of food matrices with minimal sample preparation and low cost. Moreover, smartphone-based electrochemical biosensors can be integrated with wireless communication and cloud computing to enable real-time data processing, storage, and sharing [2].

Smartphone-based electrochemical biosensors have been mainly applied to point-of-care (POC) diagnostic tools for health monitoring [3]. At the same time, still, few works in the literature are focused on food quality evaluation [4]. Analyzing the characteristics of food products is of crucial importance for the determination of their quality (e.g., pH and temperature standards [5]), nutritional value (e.g., nutrient levels [6]), authenticity (e.g., adulterants [7] detection), and safety (e.g., pathogens, allergens, contaminants detection [8]).

Currently, particular attention is given to promoting body wellness by following optimal dietary protocols specific to the individual, providing the proper micronutrient intake, and preventing chronic diseases such as diabetes, obesity, and heart diseases [9,10]. A key role is played by polyphenols (Phs), non-enzymatic exogenous antioxidants present in natural phytochemical compounds (e.g., coffee, tea, fruits) able to directly inactivate free radicals, which in the human body are responsible for the cellular oxidative stress involved in the damage of membranes, proteins, DNA, and lipids [11]. Phs show the ability to promote immunity towards foreign pathogens through numerous biological activities [12,13], reducing risks of arteriosclerosis, cancer, neurodegenerative diseases, and osteoporosis [14], as well as regulating intestinal mucosal immune responses and allergic diseases [15,16,17]. 

Coffee beverages are widely consumed worldwide [18], representing one of the principal sources of Phs in the human diet [19]. Several scientific studies directly correlate coffee intake with a lower risk of type 2 diabetes mellitus, kidney stones, Parkinson’s disease, liver cancer or fibrosis, etc. [20], making the Phs content an important index of the nutritional quality of coffee at the industrial and commercial scales. 

Phs concentration in coffee primarily depends on its variety. A hundred coffee species are available and two of them are the most cultivated in the world, “*Arabica*” (*Coffea arabica* L.) and “*Robusta*” (*Coffea canephora*) [21]. When the coffee is not roasted, it is called “green”; specifically, it is the “*Robusta*” variety, in this case, that shows the maximum Phs content (7–14.4% of the dry weight), which is higher than that for the “*Arabica*” variety (6–7% of the dry weight) [22,23]. The roasting degree of the beans is also an important parameter since both time and temperature directly affect the Phs content, as does the airflow manipulation in commercial coffee-roasting setups [24]. Green coffee has the highest range of Phs [25]. In contrast, high-temperature treatments induce Phs degradation, sugar decomposition, lipid oxidation, and pyrolysis, thus determining the color, aroma and flavor, and its bioactive compounds content. 

Typically, Phs content in coffee samples is detected in laboratories using expensive and complex instruments. Smartphone-based electrochemical biosensors offer a low-cost, portable, and user-friendly alternative for Phs detection in various settings [26]. Herein, a low-cost smartphone-based amperometric biosensor is proposed for the on-site quantification of total Phs content (TPP) in coffee blends. The device was designed for single-use and based on a previous electrochemical platform developed by our laboratory [27], where a carbon screen-printed electrode (SPE) was modified via drop-casting with multi-walled carbon nanotubes (CNTs) and gold nanoparticles (GNPs). After that, the Laccase enzyme from *Trametes versicolor* (*Tv*Lac) was immobilized through poly(vinyl alcohol)-bearing styrylpyridinium groups (PVA-SbQ) photopolymerization. In the present work, a commercial SPE already modified at the surface by CNTs-GNPs nanostructures was employed, quickly immobilizing the *Tv*Lac via glutaraldehyde (GA) cross-linking, improving the homogeneity and stability of the sensor. As is well known, this enzyme can catalyze the oxidation of ortho- and para-diphenols, aminophenols, polyphenols, polyamines, lignins, and aryl diamines [28]. The developed biosensor was optimized for *Tv*Lac and GA concentrations by monitoring the enzyme kinetic efficiency through electrochemical tests. The commercial electrode modifications were investigated by scanning electron microscopy (SEM) and Fourier-transform infrared (FT-IR) measurements, highlighting improvements in electroactive area (A_el_), surface roughness index (ρ), and also homogeneity as the *Tv*Lac is immobilized through the formation of GA cross-linked aggregates. The smartphone-based device proposed provides a sensitivity of 0.12 μA/μM, a linear response ranging between 5 and 70 μM, and a limit of detection (LOD) of 2.99 μM towards Phs content in coffee samples. The influence of the coffee variety and the roasting degree, both roasting time and temperature, were further evaluated with excellent results comparable to those obtained by the Folin–Ciocâlteu standard method. To the best of our knowledge, this was the first time, that the coupling of an electrochemical biosensor and a smartphone interface was realized for the quick determination of TPP in coffee blends, representing a possible starting point for the development of food quality screening devices with improved accessibility and user-friendliness.

## 2. Results and Discussions

*Tv*Lac is a blue multicopper oxidase enzyme (BMCO) of the oxidoreductase family, with an active site consisting of three copper centers, namely, T1Cu, T2Cu, and T3Cu. The T1Cu site is responsible for the substrate’s oxidation, while the T2Cu-T3Cu center binds the oxygen, which is converted into water through a four-electron reduction process [29]. Thus, we based our biosensor on the activity of *Tv*Lac, which can mediate the reduction of O_2_ to H_2_O through the catalytic oxidation of specific substrates such as Phs. Measuring the concentration of Phs from the cathodic current produced by the Phs(ox) reduction during the enzyme regeneration is possible. The *Tv*Lac was immobilized through GA intermolecular cross-linking and physical adsorption, with evaluation of the optimal concentration to not obstruct the substrate diffusion through the membrane and to improve the sensor stability over time.

Improvements in biosensor sensitivity and enzyme loading were obtained by testing commercial SPEs already modified with different nanomaterial combinations: (i) unmodified carbon SPE (DRP-110), (ii) CNTs modified carbon SPE (DRP-110CNT), (iii) GNPs modified carbon SPE (DRP-110GNP), and (iv) CNTs and GNPs modified carbon SPE (DRP-110CNT-GNP).

**Figure 1:**
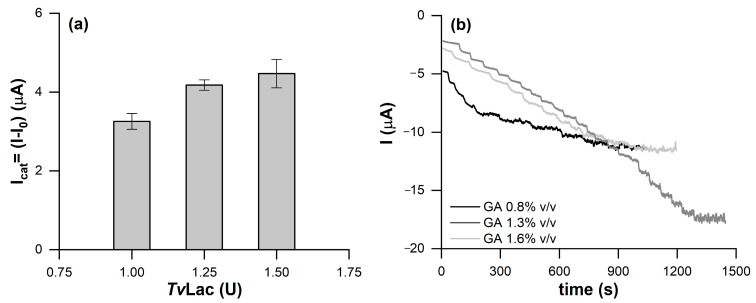
(**a**) Catalytic currents obtained through CV measurements by modifying the DRP-110GNP-CNT platform with 1, 1.25, and 1.5 U/mL of *Tv*Lac. (**b**) CA responses of the DRP-110GNP-CNT platform modified with 1.25 U of *Tv*Lac cross-linked with GA at 0.8%, 1.3%, and 1.6% *v*/*v*.

The optimal amount of *Tv*Lac to immobilize was assessed on the DRP-110CNT-GNP SPE by testing three different amounts of the enzyme: 1, 1.25, and 1.5 enzyme units (U). The catalytic current, Figure 1a, increased with the increase in the immobilized enzyme units on the working electrode (WE) surface. However, although 1.50 U provides for the highest catalytic current, 1.25 U was chosen as the optimal amount, giving a current magnitude almost comparable to that obtained with 1.50 U, but with grater reproducibility. 

GA concentration was also evaluated, namely, 0.8%, 1.3%, and 1.6% *v*/*v*. Chronoamperometry tests (CA) were performed by applying a constant potential of −0.1 V vs. a silver pseudo-reference electrode (AgPRE). As shown in Figure 1b, 0.8% *v*/*v* GA was responsible for less stability of the current over time, losing the CA response at 6 μM of catechol. This was probably due to a weaker cross-linking, exposing more of the enzyme to the solution and eventually inducing bioreceptor loss.

The biosensors modified with GA at 1.3% and 1.6% *v*/*v* showed comparable responses towards the substrate; however, in the case of GA at 1.6% *v*/*v*, the stronger cross-linking produced a denser GA matrix, hindering the substrate diffusion and causing the saturation of the signal at a catechol concentration of 20 μM. The 1.3% *v*/*v* GA solution was selected as optimal, ensuring a wider concentration range of analysis (0–2000 μM) and good sensitivity.

**Figure 2:**
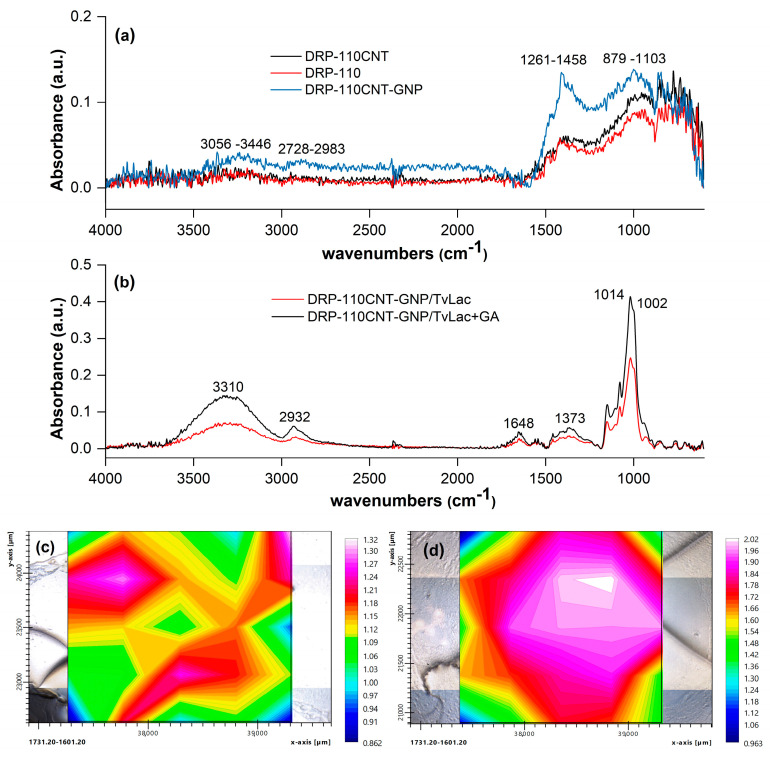
Surface analysis of electrodes: FT-IR spectra obtained for the different platforms: (**a**) DRP-110, DRP-110CNT, and DRP-110CNT-GNP; (**b**) DRP-110CNT-GNP/*Tv*Lac and DRP-110CNT-GNP/*Tv*Lac + GA; the comparison among the spectra is related to the grid central spot. Distribution maps of the CO–NH amide I bond: (**c**) integration of the band 1601.20–1731.20 cm^−1^ over the DRP-110CNT-GNP/*Tv*Lac surface; (**d**) integration of the band 1601.20–1731.20 cm^−1^ over the DRP-110CNT-GNP/*Tv*Lac + GA surface.

The surface modification of SPE electrodes was investigated through FT-IR/imaging analysis (Figure 2) and scanning electron microscopy (SEM) (Figure S1). Specifically, FT-IR measurements were performed by collecting 25 different spots in a 5 × 5 grid array area on the electrode surface (25 pixels). Figure 2a reports the FT-IR responses of DRP-110, DRP-110CNT, and DRP-110CNT-GNP electrodes at a central grid spot. The DRP-110 electrode showed two characteristic broad bands between 879 and 1103 cm^−1^ and between 1261 and 1458 cm^−1^, corresponding to the alkoxy C–O stretching and C–C stretching, respectively. After introducing CNTs at the electrode surface, there was a slight increase in the aforementioned bands, while an additional presence of GNPs enhanced the absorbance response, inducing a surface IR enhancement effect (SEIRS) [30]. Moreover, GNPs promoted the appearance of two broad bands at 2728–2983 cm^−1^ and 3056–3446 cm^−1^, associated with the C–H asymmetric and symmetric stretching and alcohol/carboxylic O–H stretching, respectively. 

The FT-IR profile when *Tv*Lac was immobilized on the WE surface by physical adsorption showed five characteristic bands, as seen in Figure 2b: the C–O–C asymmetric and symmetric stretching at 1014 cm^−1^ and 1002 cm^−1^, the C–N stretching at 1373 cm^−1^, the CO–NH stretching of peptide linkage at 1648 cm^−1^, the alkylic C–H stretching at 2932 cm^−1^, and the N–H/O–H stretching at 3310 cm^−1^ [31,32,33]. FT-IR tests were also performed after depositing *Tv*Lac onto the DRP-110CNT-GNP electrode. The presence of hydrophobic forces usually induces difficulty in identifying the C–O–C and the CO–NH FT-IR spectrum [32]. However, in our case, the presence of a *Tv*Lac multilayer on the electrode, where the outer protein layer had no direct interaction with the carbon-based electrode, made it possible to distinguish the vibrations of the C–O–C and CO–NH functionalities (1002–1140 cm^−1^ and 1648 cm^−1^, respectively). By integrating the area of the CO–NH stretching band over the entire electrode surface (i.e., considering the 25 different collected spectra), it was possible to map the surface distribution of *Tv*Lac, as shown in Figure 2c,d. The CO–NH stretching band was selected as the reference signal for the FT-IR imaging, being the peptide linkage present in the whole *Tv*Lac structure. In fact, choosing other functionalities present only in specific regions of the protein would only give information about the *Tv*Lac enzyme orientation with respect to the electrode surface. Since the CO–NH stretching vibration was directly influenced by the hydrophobic interaction strength between the protein and the carbon surface, the lower the amount of *Tv*Lac in a specific region, the lower the possibility of observing the CO–NH stretching band when the FT-IR spectrum was collected at that spot. Therefore, by observing the map, an irregular distribution of the CO–NH stretching vibration intensity highlighted non-homogeneous deposition of the enzyme on the electrode surface when immobilized in the absence of GA. On the other hand, when *Tv*Lac was immobilized through the formation of GA cross-linked aggregates, the intensity of FT-IR absorbance was higher. In this case, the O–H stretching vibration band was influenced by different contributions: (i) the vibration of the OH present in the protein structure; (ii) the vibration of the OH related to the incorporated water molecules; and (iii) the vibration of the OH present in the GA structure [34]. Also, the alkylic C–H stretching at 2932 cm^−1^ was due to both GA and *Tv*Lac. The remaining vibrational modes were only related to the protein; therefore, the increase in spectrum intensity was due to a higher concentration of the biomolecule in the central spot, which suggested a different distribution over the electrode surface than that previously described. This was confirmed by the distribution map shown in Figure 2d, where the CO–NH stretching mode vibration intensity is strong in the electrode surface central region, which is an index of the homogeneous immobilization of *Tv*Lac via GA cross-linking.

**Figure 3:**
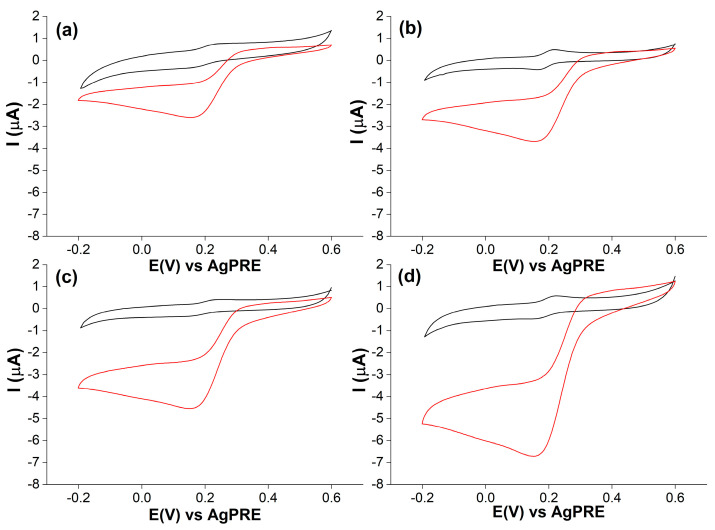
CV measurements were carried out in a solution of catechol 0.02 mM, AcONa buffer 0.01 mM, pH 5, and scan rate 5 mV/s in presence (red curves) and absence (black curves) of *Tv*Lac for (**a**) DRP-110; (**b**) DRP-110CNT; (**c**) DRP-110GNP; (**d**) DRP-110GNP-CNT.

CV tests, shown in Figure S2, were performed to calculate the electroactive area (A_el_) of the four SPEs. Specifically, CV measurements at different scan rates were performed in a solution of 1 mM [Fe (CN)6]^3−/4−^ with KCl 0.1 M. According to the Randles–Ševčík equation and the obtained plot of I_p,a_ vs. v^1/2^ [35], the A_el_ values were calculated and they are summarized in Table S1 with the relative roughness factors (ρ) defined by the electroactive/geometric area ratio (A_el_/A_geo_). Both A_el_ and ρ increased in the order DRP-110 < DRP-110GNP < DRP-110CNT < DRP-110CNT-GNP. Further CV measurements were conducted to evaluate the catalytic response of *Tv*Lac depending on the specific surface modification, as shown in Figure 3, in sodium acetate buffer (AcONa) 0.01 mM, pH 5, with catechol 0.02 mM, in the presence and absence of the enzyme. 

When the enzyme was employed, the voltammograms showed diffusion-controlled anodic and cathodic peaks for the oxidation and reduction of the catechol at the electrode surface. In the presence of *Tv*Lac, if the catalysis regularly took place, the voltammogram shapes became characteristic sigmoidal curves. Because the oxidation of the catechol was catalyzed by the enzyme, the anodic peak disappeared, while the cathodic peak became broader, indicating a higher amount of catechol reduced at the electrode surface [36]. Following the steady-state catalytic current and the sigmoidal morphology, the performance of each electrode was evaluated. The platform DRP-110 showed a cathodic current of 0.8 μA, while the additional presence of CNTs in DRP-110CNT (Figure 3a,b) induced an increase in the current to 1.7 μA, broadening the cathodic peak. The higher electronic conductivity and electroactive area provided by the presence of CNTs resulted in a faster electron transfer (ET) at the electrode surface.

**Figure 4:**
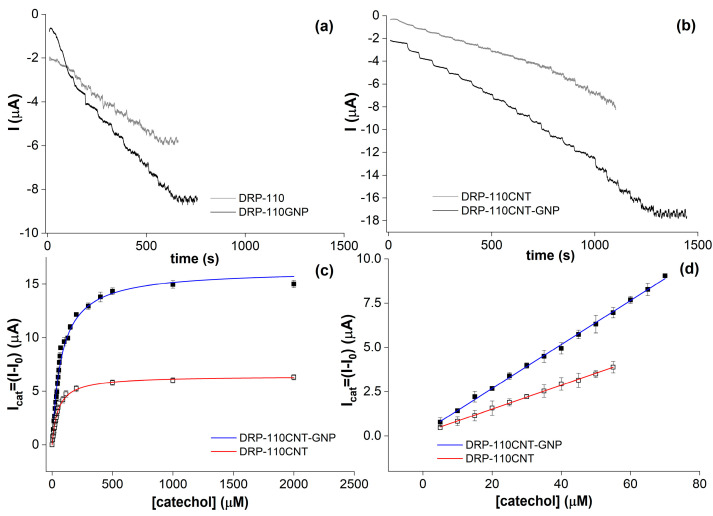
CA measurements were carried out in AcONa buffer 0.01 M, pH 5, E_dc_ = −0.1 V, and t_interval_ = 0.1 s, by adding equal volumes of a catechol solution at 255 mM, under constant stirring. CA profiles for *Tv*Lac cross-linked with GA on (**a**) DRP-110 vs. DRP-110GNP and (**b**) DRP-110CNT vs. DRP-110CNT-GNP; (**c**) Michaelis–Menten curves obtained by fitting the I_cat_ data with increasing catechol concentration related to the CA profiles reported in (**b**). Kinetic parameters: $K_{M(CNT)}^{app}$ = 49.8 μM ± 3.56, *V_MAX(CNT)_* = 6.42 μA ± 0.15, $K_{M(CNT-GNP)}^{app}$ = 77.93 μM ± 4.41, and *V_MAX(CNT-GNP)_* = 16.33 μA ± 0.32. All the standard deviations ranged between 0.2 and 0.5 μA. (**d**) Linear ranges of response for DRP-110CNT and DRP-110CNT-GNP platforms and the linear fit equations were, respectively, y = 0.16145 + 0.06753x (R^2^ = 0.9985) and y = 0.18923 + 0.12421x (R^2^ = 0.9989). All the standard deviations ranged between 0.2 and 0.5 μA.

The DRP-110GNP electrode showed an increased catalytic current of 3 μA, as seen in Figure 3c, despite the lower electroactive area compared with the DRP-110CNT electrode. This behavior was due to a stronger direct ET communication between the *Tv*Lac and the WE surface promoted by GNPs, known to work as highly efficient electron-conducting tunnels between WE and enzymes [37]. In addition, GNPs have remarkable affinity with proteins, allowing a suitable surface coverage and preserving the catalytic activity simultaneously [38,39,40,41]. By combining the GNPs and CNTs in the DRP-110CNT-GNP platform, the highest catalytic current of 4.05 μA was reached, as seen in Figure 3d. The catalytic behavior was further investigated through CA tests, carried out by measuring the cathodic catalytic current produced through successive additions of equal volumes of a catechol solution at 255 mM over time, as seen in Figure 4a,b. 

The DRP-110/*Tv*Lac + GA sensor showed the lowest signal-to-noise ratio (S/N) in the CA profile and less stability of signal over time, shown in Figure 4a in grey, as the catalytic current was constant after 20 μM of catechol, impeding the production of an acceptable Michaelis–Menten hyperbole and the kinetic parameters calculation. This behavior can be explained as a consequence of three factors: (i) immobilization of a lower amount of the enzyme because it had the smallest electroactive area and thus the lowest roughness and porosity of the unmodified carbon electrode surface (A_el_ = 2.04 mm^2^, ρ = 0.16); (ii) the consequent fast saturation of enzyme active sites; and (iii) possible enzyme loss in solution because of weaker physical adsorption on electrode material and the poor adhesion of the glutaraldehyde matrix on the same. The DRP-110GNP/*Tv*Lac + GA platform revealed better sensitivity to the substrate, as seen in the higher slope of the CA profile compared to that of DRP-110, and shown in Figure 4a in black, as highlighted before. However, there was a low stability over time of the CA signal and the S/N ratio, probably meaning that the roughness (r = 0.68) and porosity of the WE surface were unsuitable to guarantee a long permanence of the GA cross-linked aggregate. Also, in this case, the kinetic parameters could not be determined. On the other hand, the presence of CNTs in DRP-110CNT/*Tv*Lac + GA significantly favored the stability of the catalytic activity over time up to 400 mM of catechol, shown in Figure 4b in grey. The well-known high porosity of CNTs plays a dramatic role in the physical adsorption mechanism and in the immobilization stability [42,43,44,45,46]. However, the catalytic current increased slower than with the DRP-110GNP/*Tv*Lac + GA electrode, an index of lower sensitivity. The co-presence of GNPs and CNTs in DRP-110CNT-GNP/*Tv*Lac + GA, shown in Figure 4b in black, guaranteed an optimal direct ET at the surface, high enzyme loading, and good immobilization stability at the same time, providing for a sensitive and stable CA response over time. The Michaelis–Menten hyperboles were obtained for the last two platforms (Figure 4c), along with calculations of the kinetic parameters and ranges of linear response (Figure 4d) for both. 

The DRP-110CNT-GNP/*Tv*Lac + GA was chosen for the realization of the smartphone-based biosensor for Phs detection in coffee blends, with a linear range of 5–70 μM, analytical sensitivity (a) equal to 0.124 μA/μM, and a limit of detection (LOD) of 2.99 μM. The LOD was calculated from the analytical sensitivity through the following equation: LOD = kσ_B_/a, where σ_B_ is the standard deviation of the blank measurements and k is chosen relative to the confidence level required [47]; in this work k = 3. Also, the stability over time of the biosensor was evaluated, giving a 94% retention of the response after 21 days (Figure S3). CA tests were also conducted to evaluate the interference effects on the catalytic response by glucose, fructose, and sucrose, which are commonly present in coffee matrices [48] (Figure S4). The signal variation induced by these species ranged between 0 and 0.15 μA, which was in accordance with the experimental error of the biosensor. Total Phs content was evaluated in seven different coffee blends (Table S2) that differed in roasting degrees and origin. More specifically, the roasting degree influence was studied by testing a green coffee sample treated in an oven under different temperatures and times. All the coffee blends were analyzed using the biosensor device and recording a CA with a constant applied potential of −0.1 V vs. AgPRE. 

**Figure 5:**
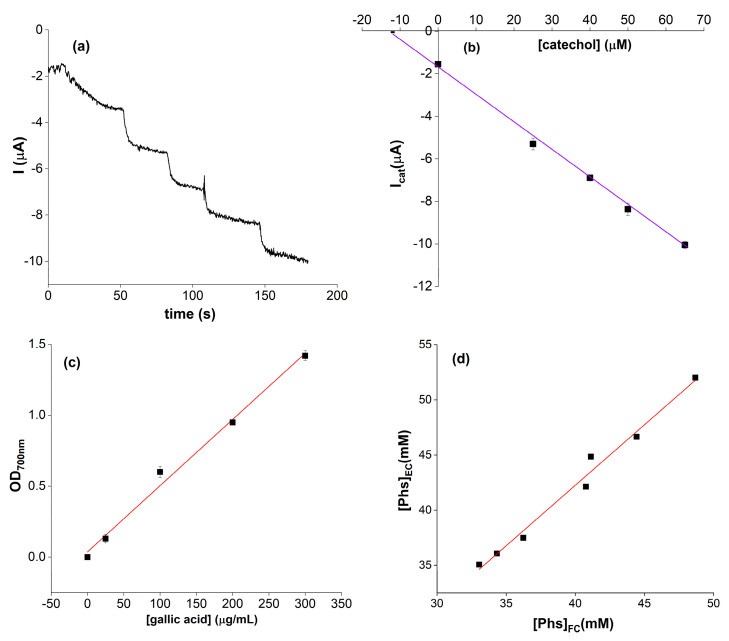
(**a**) Chronoamperogram for the analysis of coffee blend II and (**b**) the corresponding graph of standard additions; the equation of the line is y = −0.1306x − 1.7263 (R^2^ = 0.9978). The standard deviations ranged between 0.08 and 0.3. (**c**) Standard calibration line obtained with the Folin–Ciocâlteu method. The linear fit equation was y = 0.005x + 0.036 (R^2^ = 0.988). The standard deviations ranged between 0.01 and 0.04; (**d**) correlation between the results obtained through Folin–Ciocâlteu method (FC) and by the electrochemical biosensor (EC); the linear fit equation was y = 1.097x − 1.634 (R^2^ = 0.984).

The method used for the analysis was the standard addition method: the current intensity value of the blank (I_0_) was recorded and, sequentially, the unknown sample followed by the standard catechol solutions at 25, 40, 50, and 65 μM were added. As a reference, the CA profile for the analysis of coffee blend II and the corresponding graph of standard additions are shown in Figure 5a,b. Observing the chronoamperometric profile, the first step related to the addition of the real sample appeared rather different compared to the other steps. This behavior was likely due to the complexity of the real matrix containing various types of polyphenols (e.g., caffeic acid, ferulic acid, coumaric acid [49]) with different diffusion coefficients in solution depending on the pH conditions, electrolyte concentrations, and electrode surface modifications [50,51]. The I_cat_ related to each standard sample was plotted vs. the corresponding catechol standard concentration and the one related to the unknown sample corresponded in the graph to [catechol] = 0 μM. The Phs concentration was determined by extrapolation of the line passing through the points, considering the dilution factor. The reference method employed for the validation of the biosensor was the Folin–Ciocâlteu spectrophotometric assay (calibration curve in Figure 5c) obtained by diluting each sample 200:1. 

All the values of Phs content obtained with the two methods are reported in Table 1. The experimental data were finally converted into mg/g, defined as the total concentration of Phs expressed as gallic acid on the total grams (7g) of fresh coffee used to prepare the sample. The analysis of the actual samples thus led to satisfactory results in agreement with previous work in the literature [19,52], in which spectrophotometric methods determined the concentration of polyphenols.

The values obtained using the optimized biosensor agreed very well with those obtained by Folin–Ciocâlteu, with a mean recovery of 95%. The results confirmed the dependence of Phs content on the coffee variety (*C. robusta* or *C. arabica*) and the roasting degree (light, medium, dark). The roasting temperature causes chlorogenic acid degradation, reducing the amounts of malic and citric acid, and thus the quinic acid concentration [53,54]. Specifically, by considering the blends I, II, and III, which were characterized by the same variety (100% *C. arabica*), a higher roasting degree led to greater degradation of Phs, resulting in a lower content. The samples IV, V, and VI, belonging to the same manufacturer, were characterized by different percentages of *C. arabica* and *C. robusta*.

The highest Phs concentrations were found in coffee blends with higher amounts of *C. robusta*, as has been reported in the literature [55,56]. The samples VI and II, belonging to different manufacturers, showed similar Phs concentrations, being characterized by a medium roasting degree and a composition of 100% *C. arabica*. Interestingly, the case of coffee blend VII, which was composed of a blend of 98% of a *C. arabica* variety and 2% of *C. robusta* green, presented a higher Phs content than blends II and I, but it was similar to blend III; in fact, the presence of green coffee in a very low percentage did not significantly influence the Phs content. The last blend analyzed was VIII, a green coffee 100% *C. arabica*, roasted under different temperatures and time conditions. The results reported in Table 2, besides being in good agreement with those obtained through the Folin–Ciocâlteu reference method, were also consistent with the behaviors widely treated in the literature [52,57].

The lightly roasted samples generally showed the highest Phs content [58], compared to the relatively unroasted samples. In fact, thermal treatments were responsible for both the degradation of antioxidants and the production of new species such as heterocyclic compounds obtained through the Maillard reaction. However, as the breakdown of cellular components during thermal processes is responsible for the release of bound phenolic acids, the higher the roasting time, the lower the total Phs content was under all roasting temperatures. 

Finally, the results obtained with the biosensors were correlated with those of the Folin–Ciocoalteu method (Figure 5d), to evaluate the reliability of the sensor. The correlation value between the two methods was found to be 0.984. 

## 3. Materials and Method

### 3.1. Reagents and Samples

Catechol, potassium chloride (KCl), sodium acetate (AcONa), sodium phosphate dibasic, sodium phosphate monobasic, glutaraldehyde 25% *v*/*v*, and laccase from *Trametes versicolor* (*Tv*Lac) were purchased from Merck Life Science (Milan, Italy). All solutions were prepared using Milli-Q water (R = 18.2 MΩ cm at 25 °C; TOC < 10 μg L^−1^, Millipore, Molsheim, France). *Tv*Lac was solubilized in sodium acetate buffer (AcONa) 0.01 M, pH 5, stored at −20 °C, and catechol standard solutions were freshly prepared in AcONa buffer 0.01 M, pH 5, for each experiment. 

### 3.2. Electrochemical and Surface Characterization Apparatus

**Figure 6:**
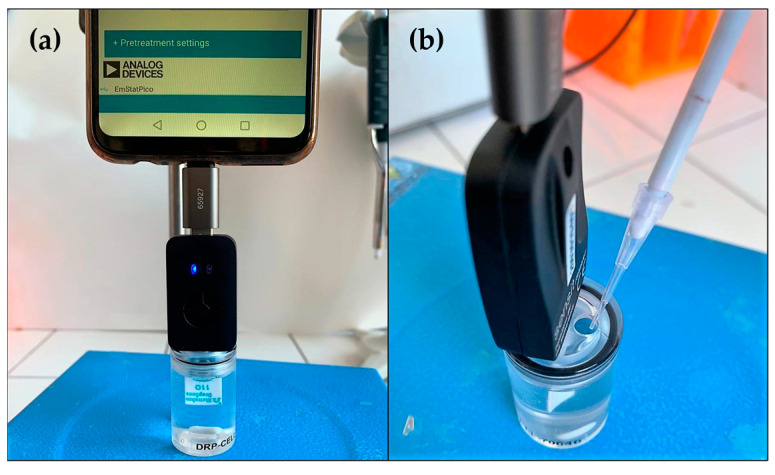
(**a**) Measurement setup; (**b**) catechol solution addition.

All the CV and CA measurements were performed by using a portable potentiostat, the Sensit Smart potentiostat by PalmSens, controlled by PSTouch on a smartphone. The experiments were carried out through the three-electrode miniaturized cell of a screen-printed electrode (SPE), with a pseudo-reference electrode (PRE) made of silver and an auxiliary electrode (AE) made of carbon ink in a 7 mL electrochemical cell at RT (Figure 6a,b). The determination of the currents at high catechol concentrations was carried out with the ausilium by using an FTT filter. 

Four commercial SPEs with different working electrodes (WEs) were tested: carbon (DRP-110), carbon modified with GNPs (DRP-110GNP), carbon modified with CNTs (DRP-110CNT), and carbon modified with CNTs and GNPs (DRP-110CNT-GNP). All the SPEs were purchased already modified by Metrohm Italiana (Formello, Italy), having a WE with a diameter of 4 mm.

Scanning electron microscopy (SEM) was performed to characterize the electrode surface morphology using a Dual Beam Auriga Zeiss instrument located at the Sapienza Nanoscience & Nanotechnology Labs (SNN-Lab). The chemical imaging for spatially resolving the chemical properties of the different platforms used in this work was performed through FT-IR measurements using a Bruker Lumos II microscope.

### 3.3. Real Sample Preparation and Treatment

Eight coffee blends with a medium-fine grind for bar machines belonging to different brands were analyzed (Table S2). The effect of roasting on polyphenolic content was assessed by analyzing three coffee blends (blend I, II, and III) purchased in Rome at a historically famous artisanal roasting and blending facility, which contained selections of the best *arabica* coffee qualities from four different countries (Brazil, Vietnam, Colombia, and Indonesia). The first coffee blend (blend I) was dark roasted (240–250 °C), coffee blend II was medium roasted (210–220 °C), and coffee blend III was clear roasted (180–205 °C). The different polyphenolic content in *Coffea arabica* and *Coffea canephora* (also called *C. robusta*) varieties was assessed by testing three coffee blends (blends IV, V, and VI) obtained by blending *arabica* and *robusta* varieties in different percentages but maintaining the same medium roasting degree (210–220 °C). The values described in the literature for total polyphenols may vary from 4 to 8.4% for *C. arabica*, and from 7 to 14.4% for *C. robusta* [59]. The *C. arabica* variety contains a lower polyphenols content, prefers high-altitude cultivation between 1000 and 2000 m, and comes from global producers such as Brazil, Vietnam, Colombia, and Indonesia; *C. robusta*, on the other hand, has a higher polyphenols content, grows at altitudes below 700 m, and comes from Mexico, Guatemala, Honduras, Nicaragua, El Salvador, Ethiopia, India, and Ecuador [60]. Coffee blend IV was medium roasted and the coffee was obtained from a skillful blend of both varieties, 80% *C. robusta,* which gives body and intensity, and 20% *C. arabica,* with a fine and characteristic aroma. Coffee blend V was 40% *C. arabica* and 60% *C. robusta*. Finally, coffee blend VI was 100% *arabica*, obtained from a careful blending of the best selections of coffee from Central and South America, Africa, and East Asia. Moreover, a blend of green coffee obtained from ‘raw’ coffee beans which had not undergone the roasting process but only the drying process was also analyzed. The coffee obtained contained a high-quality polyphenol chlorogenic acid, which is generally reduced during the roasting process [61]. Coffee blend VII (98% *arabica*, 2% *robusta* green) came from a small community of native Indians in Central America. Green coffee (blend VIII, 100% *arabica*) was then roasted in the laboratory oven at three different temperatures (180, 200, and 250 °C) to examine the impact of roasting on polyphenol concentration. Every sample was taken off the oven at regular temperature intervals (10, 15, and 20 min) to obtain coffee beans with various roasting levels.

Coffee making refers to the coffee-making standard for sensory analysis in ISO 6668:2008, where as much as 7 g of brewed coffee is used in 100 mL of boiling water of 92–96 °C with an unfiltered pour-over method. After extraction (3 min), the samples were filtered through a Whatman5 paper filter 15 cm in diameter (Vetroscientifica srl, Rome, Italy). Finally, cooled samples were diluted 1:3000 with 0.01 M AcONa buffer, pH = 5, for the electrochemical tests, or with 0.01 PBS, pH = 7.4, for the Folin–Ciocâlteu assay.

### 3.4. Folin–Ciocâlteu Assay 

As a reference method to measure the total polyphenol content in coffee samples, the Folin–Ciocâlteu method was employed. This colorimetric assay consists of using the Folin–Ciocâlteu reagent, composed of phosphomolybdate and phosphotungstate, which can react with phenolic compounds in an alkaline medium, causing a change in color which is proportional to the concentration of phenolic compounds in the sample [62]. The determination of the total polyphenol content is expressed through gallic acid equivalents. For this purpose, a calibration curve was made with standard solutions of gallic acid prepared at known concentrations (0, 25, 100, and 300 µg/mL) using the BioQuoChem^®^ (Asturias, Spain) kit for the spectrophotometric determination. Specifically, 500 µL of the Folin–Ciocâlteu reagent and 400 µL of a carbonate and hydrogen carbonate buffer solution were added to 100 µL of each standard solution for a total volume of 1 mL [63]. The obtained solutions were mixed and incubated for 15 min at room temperature. The absorbance of standard solutions in quartz cuvettes was measured by a T60U (UV-Visible) Spectrophotometer from PG Instruments Ltd. at a wavelength of 700 nm. The same procedure was followed with the coffee samples of unknown polyphenol concentration, previously diluted 1:3000 with PBS 0.01 M, pH 7.4, so that the absorbance at 700 nm fell within the limits of the standard curve.

### 3.5. Specificity Studies

Specificity studies were carried out via CA tests, measuring the catalytic current produced when 40 μM catechol was added, in the presence and absence of glucose, fructose, and sucrose at the ratio catechol:sugar/1:0.5.

### 3.6. TvLac-Based Biosensor Fabrication

Each SPE was easily modified by drop casting of a solution composed of 2 μL of GA 8% *v*/*v* and 10 μL of *Tv*Lac 125 U/mL, to have final concentrations of GA and *Tv*Lac of 1.33% *v*/*v* and 1.25 U/mL, respectively. Afterward, the electrode was left in the refrigerator at 4° C for 60 min to promote a stable intermolecular cross-linking among the enzyme molecules [64,65] and then left drying for 30 min at RT, allowing the physical adsorption of the cross-linked laccase molecules onto the WE surface. 

### 3.7. Electrochemical Measurements 

CV measurements were carried out in 7 mL of catechol 0.02 mM, prepared in AcONa buffer 10 mM, pH = 5, in the presence and absence of the enzyme, from 0.6 V to −0.2 V vs. AgPRE, scan rate 5 mV/s. The CA measurements were carried out under constant stirring by applying a fixed potential equal to −0.1 V to the WE according to the literature [27,36,66], in 7 mL of AcONa buffer solution 10 mM, pH = 5, and by adding equal volumes of 75 mM concentrated catechol solution, prepared in AcONa buffer 10 mM, pH = 5. All the experiments were carried out at RT.

## 4. Conclusions

In this work, a smartphone-based electrochemical biosensor for the rapid, sensitive, and user-friendly determination of TPP in coffee blends was realized for the first time. The use of an optimized concentration of GA in *Tv*Lac immobilization allowed a suitable ET connection with the WE and system stability over time. FT-IR imaging proved the increased homogeneity of *Tv*Lac on the sensor surface when cross-linked with GA, with a biosensor signal retention of 94% after 21 days. The calibration of the device by CA, using catechol as a standard solution, gave a linearity response ranging between 5 and 70 μM, with a lower detection limit of 2.99 μM and sensitivity at 0.124 μA/μM. By testing eight coffee samples with different roasting degrees and blends, the sensor provided results in good agreement with those obtained through the Foline–Ciocâlteu reference method, with a mean recovery of 95% and an optimal correlation value between the two methods of 0.984. One of the main advantages of using a biosensor coupled with a smartphone interface is that it eliminates the need for cumbersome and time-consuming procedures such as setting up complex instruments. Moreover, it enhances the accessibility, real-time data transmission, cost-effectiveness, and user-friendliness of such devices, making them suitable for various applications.

## Figures and Tables

**Table 1:** Polyphenols recovery in different coffee blends (*a.* = *arabica*; *r.* = *robusta*).

Coffee Blend	Variety	Roasting Degree	[Phs] (mM) Folin–Cioc.	[Phs] (mM) Biosensor	[Phs] (mg/g) Biosensor	Recovery
I	100% *a.*	Dark	33.0	35.1 ± 0.3	170.0 ± 1.5	94%
II	100% *a.*	Medium	36.2	37.5 ± 0.2	180.2 ± 1.2	96%
III	100% *a.*	Light	41.1	44.8 ± 0.5	210.8 ± 2.3	91%
IV	80% *r*. 20% *a.*	Medium	49.7	52.0 ± 0.3	250.3 ± 1.3	95%
V	60% *r.* 40% *a.*	Medium	44.4	46.6 ± 0.3	227.7 ± 1.5	95%
VI	100% *a.*	Medium	34.3	36.1 ± 0.2	170.5 ± 1.0	95%
VII	98% *a*. 2% *r.*	Medium	40.8	42.1 ± 0.2	200.5 ± 0.8	97%

**Table 2:** Polyphenols recovery in different roasted coffee blends.

Roasting Degree	Oven t (°C)	Roasting Time (min)	[Phs] (mM) Folin–Cioc.	[Phs] (mM) Biosensor	[Phs] (mg/g) Biosensor	Recovery
unroasted	/	/	22.7	22.0 ± 0.3	105.0 ± 0.3	97%
Light	180	10	32.9	31.4 ± 0.1	152.6 ± 0.5	95%
Light	180	15	16.2	17.0 ± 0.1	82.6 ± 0.6	95%
Light	180	20	8.8	9.3 ± 0.2	45.4 ± 1.0	94%
Medium	200	10	24.2	25.5 ± 0.1	124.0 ± 0.5	95%
Medium	200	15	16.9	18.1 ± 0.2	80.8 ± 1.1	93%
Medium	200	20	9.9	9.1 ± 0.1	44.4 ± 0.7	93%
Dark	250	10	16.9	18.1 ± 0.2	80.8 ± 1.1	93%
Dark	250	15	11.1	10.3 ± 0.2	50.0 ± 0.9	93%
Dark	250	20	9.7	10.0 ± 0.2	40.9 ± 1.0	96%

## Data Availability

No new data were created or analyzed in this study. Data sharing is not applicable to this article.

## Supplementary Materials

The following supporting information can be downloaded at: https://www.mdpi.com/article/10.3390/molecules28145425/s1, Figure S1. SEM images. (A) DRP-110; (B) DRP-110CNT; (C) and (D) DRP-110CNT-GNP. Figure S2. CV at different scan rates (5–1000 mV/s) in a solution of 1 mM [Fe (CN)6]^3−/4−^ with KCl 0.1 M for the calculation of the electroactive area (A_el_) of DRP-110, DRP-110GNP, DRP-110CNT, DRP-110-GNP. Figure S3. Catalytic currents obtained through CV measurements for the DRP-110GNP-CNT/*Tv*Lac/GA platform over 21 days. Figure S4. Catalytic current obtained through CA tests after the addition of 40 mM catechol alone and in presence of different interferents species at catechol:sugar/1:0.5. Table S1. A_el_ and r values for the DRP-110, DRP-110GNP, DRP-110CNT and DRP-110CNT-GNP platforms. Table S2. Polyphenols recovery in different roasted coffee blends.

## Sample Availability

Coffee samples are available from the authors.
