# Supplementary material for: Smartphone-Based Electrochemical Biosensor for On-Site Nutritional Quality Assessment of Coffee Blends
Source: Molecules. 2023 Jul 15;28(14):5425. doi: 10.3390/molecules28145425 (PMC10386176; doi:10.3390/molecules28145425)
Supplement: Supplementary file 1 [file molecules-28-05425-s001.zip › molecules-2488862-supplementary.pdf]

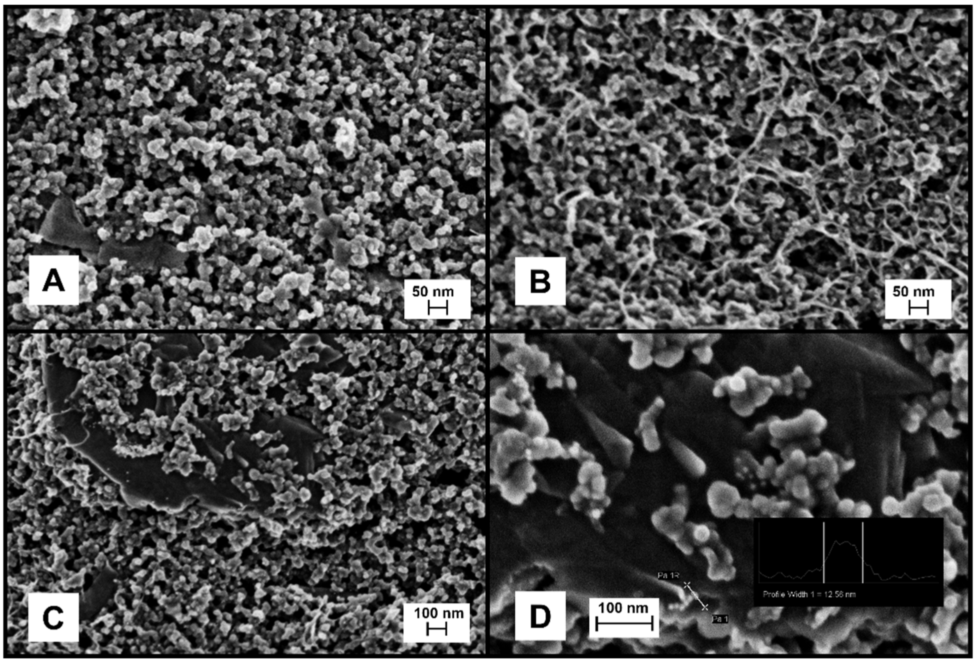

Figure S1. SEM images. **A**) DRP-110; **B**) DRP-110CNT; **C**) and **D**) DRP-110CNT-GNP.

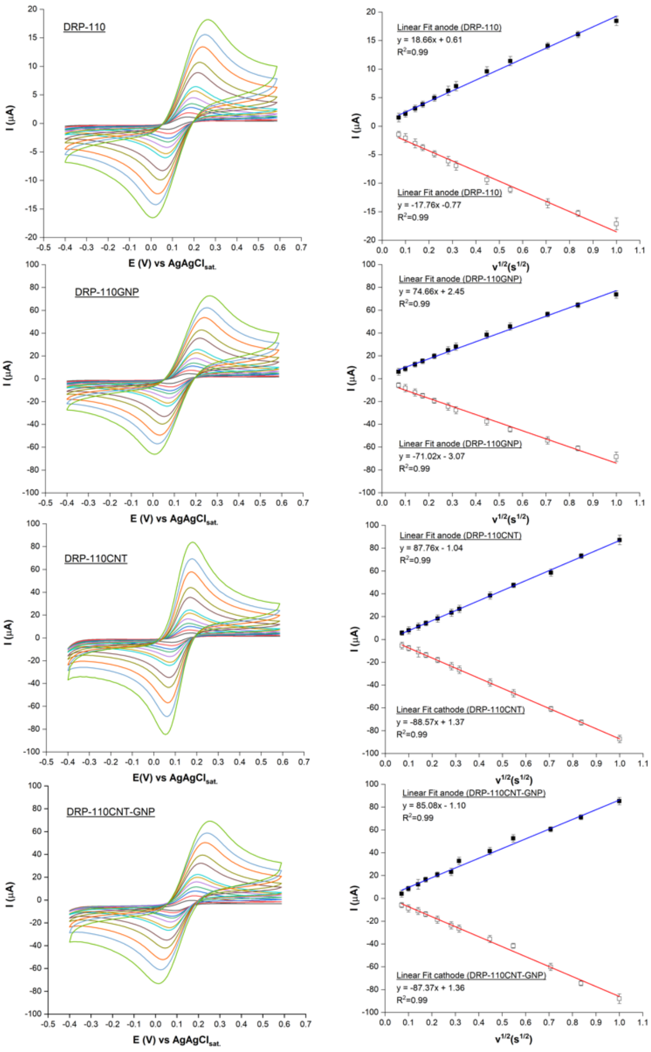

Figure S2. CV at different scan rates (5-1000 mV/s) in a solution of 1 mM [Fe (CN)6]$^{3-/4-}$ with KCl 0.1 M for the calculation of the electroactive area (A_{el}) of DRP-110, DRP-110GNP, DRP-110CNT, DRP-110-GNP.

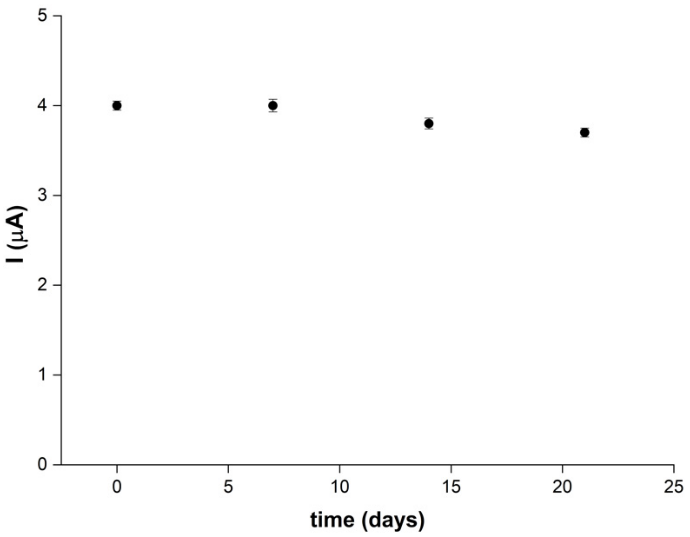

Figure S3. Catalytic currents obtained through CV measurements for the DRP-110GNP-CNT/*Tv*Lac/GA platform over 21 days.

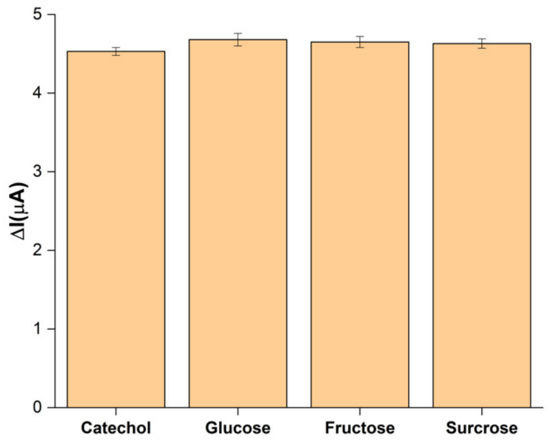

Figure S4. Catalytic current obtained through CA tests after the addition of 40 μM catechol alone and in presence of different interferents species at catechol:sugar/1:0.5.

Table S1. A_{el} and r values for the DRP-110, DRP-110GNP, DRP-110CNT and DRP-110CNT-GNP platforms.

Platform	A_{el} (mm^2)	ρ (A_{el}/A_{geo})
DRP-110	2.04 ± 0.02	0.16
DRP-110GNP	8.59 ± 0.05	0.68
DRP-110CNT	11.04 ± 0.10	0.87
DRP-110CNT-GNP	11.46 ± 0.04	0.91

Table S2. Polyphenols recovery in different roasted coffee blends.

Coffee blend sample	Coffee species	Roasting degree
I	*C. arabica* (100%)	Dark
II	*C. arabica* (100%)	Medium
III	*C. arabica* (100%)	Light
IV	*C.robusta* (80%) *C. arabica* (20%)	Medium
V	*C.robusta* (60%) *C. arabica* (40%)	Medium
VI	*C. arabica* (100%)	Medium
VII	*C. arabica* (98%) *C. robusta* (2%) green	Medium
VIII	*C.arabica (100%)* green	unroasted
